# Associations between allostatic load and hepatic steatosis and liver fibrosis: evidence from NHANES 2017–2020

**DOI:** 10.1186/s12889-024-19111-7

**Published:** 2024-06-15

**Authors:** Zhikun Dai, Xiaohui Zhou

**Affiliations:** Department of Infectious Diseases, The First Affiliated Hospital of Shantou University Medical College, Shantou , Guangdong, 515041 China

**Keywords:** Allostatic Load, Hepatic Steatosis, Liver Fibrosis, NHANES

## Abstract

**Background:**

Allostatic load, the cumulative strain resulting from chronic stress responses, has been linked to disease occurrence and progression, yet research quantifying this relationship is limited. This study aimed to explore the relationship between allostatic load score (ALS) levels and the degree of hepatic steatosis and fibrosis.

**Methods:**

Data from the National Health and Nutrition Examination Survey 2017–2020 were analyzed. The ALS was based on the statistical distribution, assigning one point for each biomarker if it was in the highest risk quartile, and then summing them to generate the ALS score (range, 0–8). The multivariate linear regression was employed to analyze the association between the controlled attenuation parameter (CAP) and liver stiffness measurement (LSM) with ALS. Additionally, multinomial logistic regression was used to investigate the association between ALS and the degree of hepatic steatosis and fibrosis.

**Results:**

Participants had a weighted mean age of 52.69 years and 56.14% were female. In the multivariate linear regression analysis, ALS showed a significant positive correlation with CAP (β = 15.56, 95% CI: 14.50–16.62) and LSM (β = 0.58, 95% CI: 0.48–0.67). Age, healthy dietary level, and PIR had significant interactions with this positive correlation. In the multinomial logistic regression analysis, ALS exhibited a significant positive correlation with different degrees of hepatic steatosis and fibrosis. Consistency of the results was observed in sensitivity analyses using clinical thresholds of ALS.

**Conclusions:**

Comprehensive clinical assessment targeting load adaptation may enhance the effectiveness of risk assessment in patients with hepatic steatosis and fibrosis.

## Introduction

Steatotic liver disease (SLD) represents a chronic hepatic disorder that afflicts over a quarter of the global population, with its incidence rapidly increasing [1, 2]. The persistence of inflammation and subsequent fibrogenesis can contribute to the progression of detrimental outcomes, including liver cirrhosis, hepatocellular carcinoma, and end-stage liver disease [3], thereby imposing a considerable strain on both individual well-being and the healthcare system. The implementation of transient elastography, extensively employed in the screening of SLD due to its precision and non-invasive characteristics, utilizes controlled attenuation parameter (CAP) and liver stiffness measurement (LSM) individually for the assessment of hepatic steatosis and fibrosis [4, 5].

Allostasis represents the extent of adaptation required in living systems, encompassing scenarios where individuals anticipate the necessity for adjustments, deviate from physiological set points, and establish new ones [6]. The notion of allostatic load, introduced by McEwen and Stellar in 1993 [7], highlights the aggregate burden of adversities faced in life and persistent stress. However, this concept surpasses conventional definitions of chronic stress, as it encompasses the progression of disease beyond such conditions. When an individual's adaptive capacity is overwhelmed by the burdens endured due to extended exposure to fluctuating or intensified neural or neuroendocrine reactions, allostatic overload may manifest [7, 8]. Employing allostatic load score (ALS), the effects of stress on the cardiovascular, metabolic, and immune systems can be quantified, establishing a reliable means to evaluate physiological reactivity towards stressors [9]. Studies have consistently demonstrated a significant correlation between elevated levels of allostatic load and a diverse range of adverse physical and mental health consequences [10, 11], and the allostatic load index predicts mortality rate and physiological function more consistently than its constituent components [11].

From an allostatic standpoint, SLD frequently coexists with chronic diseases characterized by the same metabolic disruptions or distinct underlying causes, such as diabetes and chronic hepatitis B infection. These comorbidities have the potential to modify the occurrence and course of SLD by constraining adaptive responses that are designed to decrease the risk of developing liver cancer [12]. Furthermore, recent investigations have shown that persistent psychological and physiological stress induce protracted hyperactivation and dysregulation within the body's neuroendocrine and immune systems, culminating in disruptions to glucose and lipid metabolism alongside chronic hepatic inflammatory responsiveness [13]. Evidence from cell, animal, and clinical studies suggests that high cortisol levels and psychosocial stress may be involved in SLD and liver fibrosis onset and progression [13, 14]. However, the relationship between ALS and the degree of hepatic steatosis and fibrosis remains unclear. Although prior studies have demonstrated the pervasiveness of stress in the aetiopathogenesis and treatment of SLD, we have advanced the field by identifying early stress states through ALS and thereby intervening in the psychosocial chronic stress response for more favorable and longer-lasting treatment outcomes.

Therefore, our study aims to investigate the association between ALS and hepatic steatosis and liver fibrosis in adults, utilizing a large sample of people from the National Health and Nutrition Examination Survey (NHANES) 2017–2020.

## Methods

### Data source

The National Health and Nutrition Examination Survey (NHANES), conducted by the National Center for Health Statistics (NCHS), is a nationally representative survey of the U.S. population that utilizes complex, multi-stage, and probability sampling methods to assess the health and nutritional status of the general population. The ethical approval for the administration of NHANES, including its strict procedures and protocols, was granted by the NCHS Research Ethics Review Board. Written informed consent was obtained from all participants.

### Study population

**Fig. 1:**
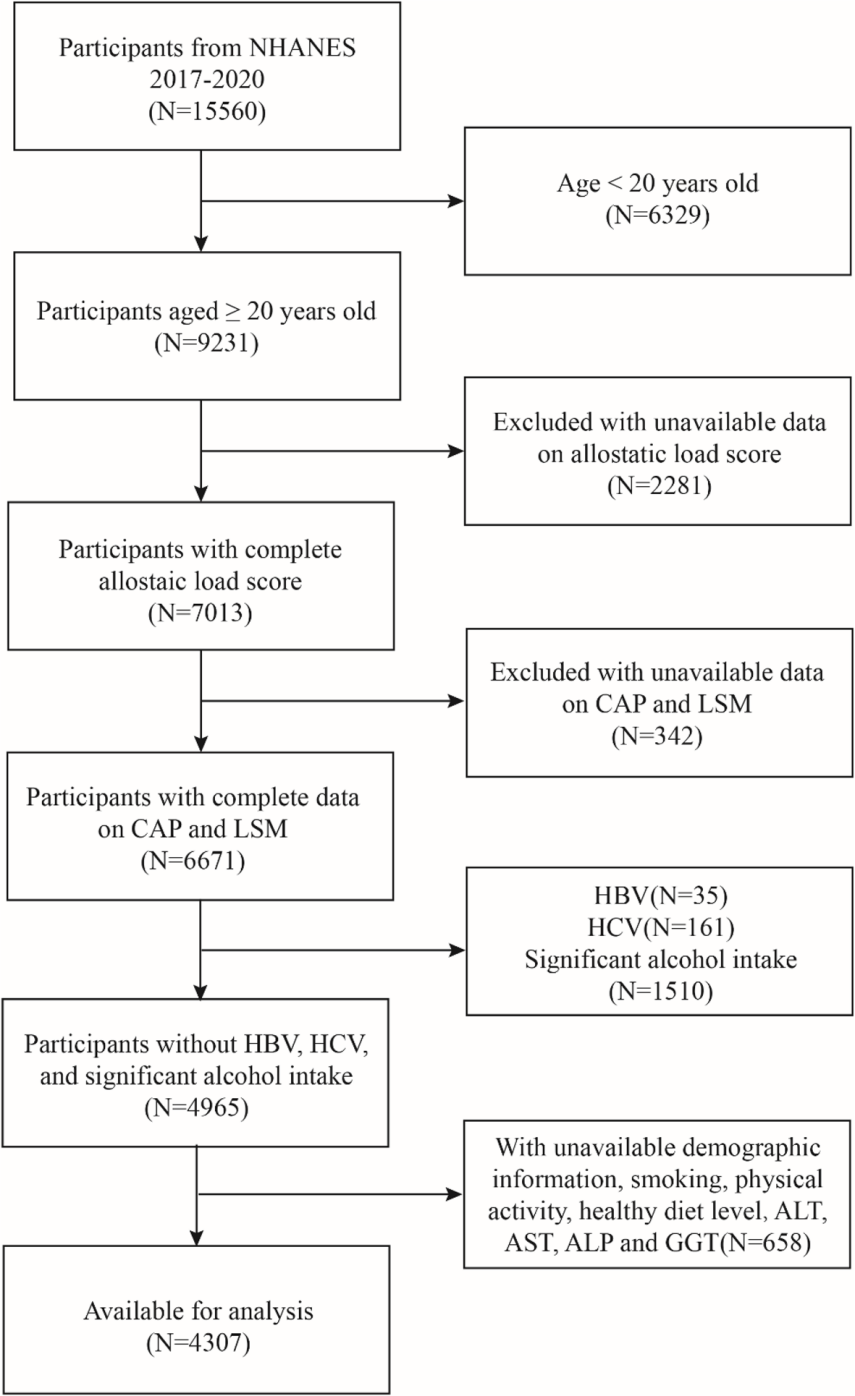
Flow Diagram of the Screening and Enrollment of Study Participants

This study utilizes data from participants in the NHANES 2017–2020 cycle as the basis for analysis. We excluded 6,329 individuals aged below 20 years from the 15,560 eligible individuals, 2,281 individuals who lacked any missing biomarkers necessary for ALS calculation, 342 subjects with missing data for CAP or LSM, 35 subjects with positive hepatitis B antigen, 161 subjects with positive hepatitis C antibody or hepatitis C RNA samples, 1,510 significant alcohol drinkers (defined as consuming more than 3 drinks a day for men and 2 drinks a day for women), 658 individuals without demographic details (including age, sex, race/ethnicity, education level, poverty income ratio (PIR), and marital status), and individuals without information on smoking, physical activity, healthy diet level, ALT, AST, GGT, and ALP. After applying these exclusion criteria, a total of 4,307 participants were selected in the analysis (Fig. 1).

### Definition of hepatic steatosis and liver fibrosis

This study relied on data obtained from the most recent NHANES 2017–2020 iteration, which implemented techniques such as ultrasound and vibration-controlled transient elastography (VCTE) to evaluate liver function. The measurements of elastography were conducted at the NHANES Mobile Examination Center using the FibroScan model 502 V2 Touch, which was equipped with either a medium (M) or extra-large (XL) wand (probe). Hepatic steatosis was determined using the median Controlled Attenuation Parameter (CAP), while liver fibrosis was assessed using the median Liver Stiffness Measurement (LSM).

Steatotic liver disease is an overarching term chosen in the latest multi-society Delphi consensus to encompass the various causes that can lead to hepatic steatosis [15], including metabolic dysfunction-associated steatotic liver disease (MASLD), metabolic alcohol-related liver disease (MetALD), alcohol-related Liver Disease (ALD), cryptogenic SLD, and specific etiologies of SLD (e.g., drug-induced, genetic, etc.).

Based on previous research findings, median CAP values of ≥ 274 dB/m and ≥ 302 dB/m have been identified as indicative of hepatic steatosis [4] and severe hepatic steatosis [1, 16], respectively. The assessment of fibrosis grades relies on liver stiffness, with critical thresholds of 8.2 kPa, 9.7 kPa, and 13.4 kPa established for fibrosis grades F2, F3, and F4, employing the Jorden index for optimization [1, 17]. Subsequently, based on the LSM grades, the participants were divided into four categories: non-fibrosis group (LSM < 8.2), significant fibrosis group (8.2 ≤ LSM < 9.7), advanced fibrosis group (9.7 ≤ LSM < 13.6), and cirrhosis group (LSM ≥ 13.6).

### Definition of allostatic load score

The array of biological markers comprising ALS in this study derive from the cardiovascular system, metabolic system, and immune system, encompassing systolic and diastolic blood pressure, total serum cholesterol (Tc), high-density lipoprotein (HDL) cholesterol, glycated hemoglobin (HbA1c), albumin (Alb), body mass index (BMI), and C-reactive protein (CRP) levels. Concerning the variables selected for ALS calculation, each investigator was required to utilize previously published ALS equations. This necessity arose from the absence of universally acknowledged clinical guidelines and the acknowledged variability in individual biomarker thresholds across different racial populations. These biomarkers chosen by us have been extensively employed in previous NHANES investigations evaluating ALS [18–20], attesting to their representation of specific organ systems' functionalities.

Regarding our analytical approach, we stratified the ALS biomarkers into quartiles based on their distributions in our database [9]. Subsequently, we converted these markers into binary variables. While albumin and high-density lipoprotein cholesterol levels are deemed high-risk if they fall within the lowest quartile, the remaining biomarkers represent high risk when they reside in the highest quartile. If a biomarker falls within the high-risk range, it is allocated a value of 1; otherwise, it receives a value of 0. The potential ALS ranges from 0 to 8, and a higher score corresponds to a stronger correlation between stress and physiological imbalances.

### Covariates

Based on previous research and clinical judgment 21, 22], the variables with gender, age, race/ethnicity, education level, marital status, poverty income ratio (PIR), healthy diet level, smoking status, activity status, ALT, AST, GGT, and ALP were established as covariates. Gender differences exist in terms of hormonal levels and responses to chronic stress. The process of aging is associated with an increased susceptibility to chronic stress and a reduction in liver function [23], thus age was evaluated from birth until baseline and categorized into 20–39, 40–59, and ≥ 60. Race/ethnicity data were collected via self-reporting and segmented into five classifications: Mexican American, non-Hispanic Black, non-Hispanic White, Other Hispanic, and Other (comprising participants of multiple races). Educational attainment may act as a protective factor or a risk factor. Those with higher levels of education may have greater access to health knowledge and self-management skills, but may also be more susceptible to chronic stress due to the nature of their work. Education level was stratified as below high school, high school or equivalent, and beyond high school. The marital status of an individual may influence the efficacy of chronic stress relief through the influence of social support networks. Marital status was categorized as married, never married, or other (including widowed, divorced, and separated individuals). PIR is a significant indicator of household poverty, and economic stress represents a significant source of chronic stress. PIR is calculated as the ratio of a family's income to the appropriate poverty threshold for that family size, as determined by the U.S. Department of Health and Human Services. PIR values were partitioned into < 1.5, 1.5–3.5, and ≥ 3.5, and a larger value indicates a higher per capita family income. Smoking is a recognized stressor in humans and has been linked to liver disease, meaning that being a covariate can help to control for the effect of smoking on study outcomes. The smoking variable gauged whether an individual had smoked a minimum of 100 cigarettes throughout their lifetime and was sorted as yes or no. The level of a healthy diet may have a direct impact on liver health, and as a covariate can help explain the potential impact of different dietary patterns on hepatic steatosis and liver fibrosis. Healthy diet level was self-reported and grouped as excellent and good, fair, and poor. Activity level was determined by whether or not an individual partook in any moderate-intensity exercise, fitness, or recreational activities during a typical week and classified as yes or no. Lastly, ALT, AST, ALP, and GGT were derived from the laboratory data of NHANES. These are liver function tests whose changes reflect liver health and as covariates can help control for the impact of the liver disease process on study outcomes.

### Statistical analysis

This study employed a complex sampling design and utilized weighted methods to minimize significant fluctuations in the dataset, in line with NHANES analysis recommendations. The baseline characteristics of the study population were statistically described based on CAP and LSM subgroups. Data for continuous variables follow a normal distribution and are expressed as means and standard deviation (SD), while data for categorical variables are expressed as numbers and percentages(%). Pearsonχ2 test was used for categorical variables, while one-way analysis of variance was used for continuous variables. After adjusting for potential confounding variables, weighted multivariate linear regression models were employed to investigate the linear association between ALS and CAP and LSM, calculating beta values and a 95% confidence interval. To verify the correlation between ALS and hepatic steatosis and fibrosis, we further grouped the variables based on the CAP and LSM values to change continuous variables into categorical variables. Since the degree of hepatic steatosis and fibrosis was used as a multicategorical response variable, we further analyzed the relationship between ALS and the degree of hepatic steatosis and liver fibrosis using weighted multiple logistic regression analysis. Model 1 was the crude model without any adjustments, while model 2 adjusted for age, gender, race/ethnicity, marital status, PIR, and education level. Model 3 adjusted for all covariates.

Furthermore, subgroup-stratified models were established to assess potential interactions between ALS and CAP and LSM, considering stratification by gender, age, education level, PIR level, healthy diet level, and smoking status. In the sensitivity analysis, we further classified each biomarker based on clinical cutoff points, assigning a score of 1 if the biomarker fell within the high-risk range. The cutoff ranges were as follows: systolic blood pressure ≥ 130 mmHg, diastolic blood pressure ≥ 80 mmHg, Tc ≥ 240 mg/dL, HDL ≤ 40 mg/dL, HbA1c ≥ 6.5%, Alb ≤ 35 g/L, BMI ≥ 30 kg/m^2^, and CRP ≥ 3 mg/dL.

All analyses in this study were conducted using R, version 4.2.0 (R Project for Statistical Computing), the survey package (version 4.11), and EmpowerStats (version 4.1).

Statistical significance was established when P-value was less than 0.05.

## Results

### Baseline characteristics of participants

In this study, a total of 4307 adult participants were included based on rigorous inclusion and exclusion criteria. The subjects had an average age of 52.69 ± 17.08 years. Within these participants, males constituted 43.86% while females accounted for 56.14%. The average (SD) concentrations of CAP, LSM, and ALS were 265.96 (62.07) dB/m, 6.05 (5.69) kPa, and 2.15 (1.63), respectively.

Table 1 presents the weighted baseline characteristics of the participants stratified based on their CAP levels. Among different CAP levels, significant disparities (all* p* < 0.05) were observed in gender, age, race/ethnicity, education level, marital status, PIR, healthy dietary level, smoking status, activity level, ALT, AST, GGT, ALP, LSM, and ALS, and these disparities exhibit statistical significance (*P* < 0.05). In comparison to the non-SLD group, individuals experiencing severe hepatic steatosis exhibited a predisposition towards older age, male gender, Non-Hispanic Black ethnicity, married, lower levels of education, diminished PIR, higher smoking status, limited moderate activity, lower healthy dietary level, as well as higher levels of ASL, ALT, ALP, GGT, LSM, and ALS.

**Table 1:** Weighted characteristics of the study population based on Controlled Attenuated Parameter (CAP)

Characteristics	Non-SLD (CAP < 302) *n* = 2383	SLD (274 < = CAP < 302) *n* = 685	Sever steatosis (CAP > = 302) *n* = 1239	*P* value
Age, mean (SD), year	48.24 (17.72)	52.77 (15.70)	53.94 (15.65)	< 0.001
Age, No. (%)				< 0.001
20–39	870 (36.51)	170 (24.80)	263 (21.25)	
40–59	780 (32.75)	278 (40.61)	468 (37.75)	
≥ 60	733 (30.74)	237 (34.59)	508 (41.00)	
Gender, No. (%)				< 0.001
Males	925 (38.81)	306 (44.73)	660 (53.30)	
Females	1458 (61.19)	379(55.27)	579 (46.70)	
Race/ethnicity, No. (%)				< 0.001
Mexican American	114 (4.78)	57 (8.33)	103 (8.30)	
Non-Hispanic White	281 (11.79)	72 (10.53)	100 (8.11)	
Non-Hispanic Black	1582 (66.39)	451 (65.80)	844 (68.10)	
Other Hispanic	165 (6.94)	34 (5.01)	79 (6.41)	
Other Race	241 (10.10)	71(10.33)	113 (9.08)	
Marital status, No. (%)				< 0.001
Married	1415 (59.38)	454 (66.21)	895 (72.26)	
Never married	484 (20.31)	74 (10.82)	134 (10.80)	
Other	484 (20.31)	157 (22.97)	210 (16.94)	
Education level, No. (%)				0.006
Less than high school	223 (9.35)	55 (8.03)	117 (9.43)	
High school or equivalent	545 (22.85)	168 (24.53)	350(28.25)	
Above high school	1615 (67.80)	462 (67.44)	772 (62.32)	
PIR, No. (%)				0.002
< 1.5	471 (19.75)	114 (16.64)	261 (21.07)	
1.5–3.5	701 (29.41)	202 (29.49)	417 (33.65)	
> = 3.5	1212 (50.84)	369 (53.87)	561 (45.28)	
Smoked at least 100, No. (%)				< 0.001
Yes	844 (35.43)	219 (32.00)	512 (41.31)	
No	1539 (64.57)	466 (68.00)	727 (58.69)	
Moderate activities, No. (%)				< 0.001
Yes	1275 (53.52)	311 (45.33)	474 (38.25)	
No	1108 (46.48)	374 (54.67)	765 (61.75)	
Healthy diet level, No. (%)				< 0.001
Excellent and good	1866 (78.32)	467 (68.18)	770 (62.13)	
Fair	436 (18.30)	182 (26.57)	373 (30.10)	
Poor	81 (3.38)	36 (5.25)	96 (7.77)	
ALT (IU/L)	18.35 (11.33)	21.49 (12.11)	26.94 (16.80)	< 0.001
AST, mean (SD, IU/L	20.10 (8.90)	20.46 (7.89)	22.52 (10.37)	< 0.001
ALP, mean (SD), IU/L	72.41 (23.77)	76.09 (21.39)	80.19 (24.13)	< 0.001
GGT, mean (SD), IU/L	22.64 (43.37)	27.76 (36.56)	33.20 (28.80)	< 0.001
LSM, mean (SD), kPa	4.94 (3.40)	5.73 (4.60)	7.54 (6.83)	< 0.001
ALS, mean (SD)	1.35 (1.29)	2.01 (1.49)	3.06 (1.61)	< 0.001

Abbreviations: *ALP* alkaline phosphatase, *ALT* alanine transaminase, *ALS* allostatic load score, *AST* aspartate transaminase, *GGT* gamma glutamyl transferase, *LSM* liver stiffness measurement, *PIR* poverty income ratio, *SLD* steatotic liver disease. All means and SD for continuous variables and percentages for categorical variables were weighted

Table 2 depicts the weighted baseline characteristics of individuals stratified based on their LSM levels. Significant differences (all *p* < 0.05) were observed in several variables, including gender, age, education level, PIR, healthy dietary level, smoking status, activity level, ALT, AST, GGT, ALP, CAP, and ALS among different LSM levels. When compared to the non-fibrosis group, individuals afflicted with liver cirrhosis were more inclined towards advanced age, male, lower levels of education, decreased PIR, higher smoking status, limited moderate activity, lower healthy dietary level, as well as higher levels of ASL, ALT, ALP, GGT, CAP, and ALS.

**Table 2:** Weighted characteristics of the study population based on median Liver Stiffness Measurement (LSM)

Characteristics	Non-fibrosis (LSM < 8.2) n = 3845	Significant fibrosis (8.2 < = LSM < 9.7) n = 162	Advanced fibrosis (9.7 < = LSM < 13.6) n = 157	Cirrhosis (LSM > = 13.6) n = 143	*P* value
Age, mean (SD), year	50.12 (17.14)	53.84 (14.90)	58.01 (14.59)	52.07 (17.49)	< 0.001
Age, No. (%)					< 0.001
20–39	1209 (31.44)	30 (18.82)	26 (16.73)	39 (27.16)	
40–59	1348 (35.06)	74 (45.66)	49 (31.34)	51 (35.80)	
≥ 60	1288 (33.50)	58 (35.52)	82 (51.93)	53 (37.04)	
Gender, No. (%)					< 0.001
Males	1646 (42.80)	91 (56.15)	75 (47.84)	76 (53.12)	
Females	2199 (57.20)	71 (43.85)	82 (52.16)	67 (46.88)	
Race/ethnicity, No. (%)					0.800
Mexican American	240 (6.24)	10 (6.17)	15 (9.55)	10 (6.99)	
Non-Hispanic White	2567 (66.76)	110 (67.90)	103 (65.61)	95 (66.43)	
Non-Hispanic Black	404 (10.51)	17 (10.50)	20 (12.74)	15 (10.49)	
Other Hispanic	246 (6.49)	14 (8.64)	10 (6.37)	9 (6.29)	
Other Race	388 (10.10)	11 (6.79)	9 (5.73)	14 (9.79)	
Marital status, No. (%)					0.575
Married	2455 (63.85)	103 (63.60)	111 (70.70)	91 (63.47)	
Never married	632 (16.44)	23 (14.38)	20 (12.74)	19 (13.52)	
Other	758 (19.71)	36 (22.02)	26 (16.56)	33 (23.01)	
Education level, No. (%)					< 0.001
Less than high school	341 (8.87)	18 (11.11)	24 (15.29)	16 (10.89)	
High school or equivalent	899 (23.38)	55 (33.95)	58 (36.94)	55 (38.80)	
Above high school	2605 (67.75)	89 (54.94)	75 (48.77)	72 (50.31)	
PIR, No. (%)					< 0.001
< 1.5	754 (19.61)	27 (16.58)	38 (24.20)	28 (19.80)	
1.5–3.5	1125 (29.26)	67 (41.19)	67 (42.68)	68 (47.30)	
> = 3.5	1966 (51.13)	68 (42.23)	52 (33.12)	47 (32.90)	
Smoked at least 100, No. (%)					0.015
Yes	1380 (35.88)	61 (37.36)	76 (48.09)	59 (41.31)	
No	2465 (64.12)	101 (62.64)	81 (51.91)	84 (58.69)	
Moderate activities, No. (%)					< 0.001
Yes	1899 (49.40)	51 (31.48)	54 (34.56)	55 (38.45)	
No	1946 (50.60)	111 (68.52)	103 (65.44)	88 (61.55)	
Healthy diet level, No. (%)					< 0.001
Excellent and good	2842 (73.91)	100 (61.69)	87 (55.61)	71 (49.65)	
Fair	828 (21.53)	52 (32.18)	52 (32.96)	61 (42.66)	
Poor	175 (4.56)	10 (6.13)	18 (11.43)	11 (7.69)	
ALT, mean (SD), IU/L	20.51 (12.84)	28.78 (17.06)	26.73 (18.75)	28.23 (20.65)	< 0.001
AST, mean (SD), IU/L	20.39 (8.18)	24.19 (11.73)	24.40 (14.08)	26.54 (20.41)	< 0.001
ALP, mean (SD), IU/L	74.05 (22.75)	85.27(30.34)	82.58 (25.65)	88.63 (32.35)	< 0.001
GGT, mean (SD), IU/L	24.58 (25.73)	35.06 (28.05)	42.92 (44.28)	54.79 (65.16)	< 0.001
CAP, mean (SD), dB/m	258.35 (59.22)	303.95 (55.79)	324.00 (57.16)	325.91 (65.33)	< 0.001
ALS, mean (SD)	1.79 (1.53)	2.94 (1.68)	3.55 (1.62)	3.36 (1.44)	< 0.001

Abbreviations: *ALP*, alkaline phosphatase; *ALT*, alanine transaminase; *ALS*, allostatic load score; *AST*, aspartate transaminase; *CAP*, controlled attenuation parameter;* GGT*, gamma glutamyl transferase;* PIR*, poverty income ratio. All means and SD for continuous variables and percentages for categorical variables were weighted

### The association between als and cap and lsm

The results of a sample-weighted multivariate linear regression analysis were employed to examine the association between ALS and CAP and LSM, as demonstrated in Table 3. In the fully adjusted model (model 3), we observed a significant positive association between ALS and CAP and LSM (CAP, Model 3: β = 15.56, 95% CI: 14.50–16.62; LSM, Model 3: β = 0.58, 95% CI: 0.48–0.67), and maintained relative stability across models. This implies that chronic stress may be significantly associated with an increased risk of hepatic steatosis and fibrosis. Furthermore, this relationship remains significant when analyzing ALS using clinical cutoff point modeling (all *p* < 0.001).

**Table 3:** Association between ALS and CAP and LSM

	**Model 1**		**Model 2**		**Model 3**	
	**β(95%CI)**	***P* value**	**β(95%CI)**	***P* value**	**β(95%CI)**	***P* value**
**ALS by quartiles**						
CAP (dB/m)	18.79(17.78–19.80)	< 0.001	18.41(17.38–19.43)	< 0.001	15.56(14.50–16.62)	< 0.001
LSM (kPa)	0.68(0.59–0.77)	< 0.001	0.67(0.58–0.77)	< 0.001	0.58 (0.48–0.67)	< 0.001
**ALS by clinical cut points**						
CAP (dB/m)	21.18(20.08–22.27)	< 0.001	20.31(19.20–21.42)	< 0.001	17.05 (15.89–18.22)	< 0.001
LSM (kPa)	0.70(0.60–0.79)	< 0.001	0.66(0.56–0.76)	< 0.001	0.54(0.43–0.65)	< 0.001

Abbreviations: *ALP*, alkaline phosphatase; *ALT*, alanine transaminase; *ALS*, allostatic load score; *AST*, aspartate transaminase; *CAP*, controlled attenuation parameter; *GGT*, gamma glutamyl transferase; *LSM*, liver stiffness measurement; *PIR*, poverty income ratio. Model 1 was the crude model without adjustment for covariates. Model 2 was adjusted for age, sex, race, marital status, education level, *PIR*. Model 3 was adjusted as for model 2, additionally adjusted for *ALT*, *AST*, *GGT*, *ALP*, healthy diet level, smoking status, physical activity

Table 4 displays the results of our further weighted multinomial logistic regression analysis, where CAP and LSM were converted from continuous variables to categorical variables. When stratifying by the degree of hepatic steatosis, strong positive associations were observed between ALS and both hepatic steatosis and severe hepatic steatosis in all three models (*p* < 0.001). In the fully adjusted model (Model 3), every one-unit increase in ALS was associated with a 0.3369-fold and 1.0182-fold increased risk of SLD and severe SLD, respectively. When stratifying by the degree of liver fibrosis, strong positive associations were found between ALS and significant fibrosis, severe fibrosis, and cirrhosis in all three models (*p* < 0.001). In the fully adjusted model (Model 3), every one-unit increase in ALS had a 0.3878-fold, 0.6910-fold, and 0.6567-fold increased risk of significant fibrosis, severe fibrosis, and cirrhosis, respectively, compared to non-fibrosis. Moreover, the significance (*p* < 0.001) of all the considerable associations persevered when ALS was delineated using clinical threshold values. Abbreviations: *ALP*, alkaline phosphatase; *ALT*, alanine transaminase; *ALS*, allostatic load score;* AST*, aspartate transaminase; *CAP*, controlled attenuation parameter;* GGT*, gamma glutamyl transferase; *LSM*, liver stiffness measurement; *PIR*, poverty income ratio. Model 1 was the crude model without adjustment for covariates. Model 2 was adjusted for age, sex, race, marital status, education level, *PIR*. Model 3 was adjusted as for model 2, additionally adjusted for *ALT*, *AST*, *GGT*, *ALP*, healthy diet level, smoking status, physical activity

**Table 4:** Association between ALS and the degree of hepatic steatosis and fibrosis

	**Model 1**		**Model 2**		**Model 3**	
	**OR (95%CI)**	***P* value**	**OR (95%CI)**	***P* value**	**OR (95%CI)**	***P* value**
**CAP**						
Non-SLD	1[Reference]		1[Reference]		1[Reference]	
**ALS by quartiles**						
SLD	1.4145 (1.4140–1.4150)	< 0.001	1.413(1.4130–1.4141)	< 0.001	1.3369 (1.3364–1.3374)	< 0.001
Severe steatosis	2.1371(2.1364–2.1378)	< 0.001	2.2109(2.2102–2.2117)	< 0.001	2.0182(2.0175–2.0189)	< 0.001
**ALS by clinical cut points**						
SLD	1.6062(1.6056–1.6068)	< 0.001	1.6004(1.5998–1.6011)	< 0.001	1.5010(1.5004–1.5017)	< 0.001
Severe steatosis	2.4181(2.4173–2.4190)	< 0.001	2.4493(2.4484–2.4502)	< 0.001	2.1841(2.1833–2.1850)	< 0.001
**LSM**						
Non-fibrosis	1[Reference]		1[Reference]		1[Reference]	
**ALS by quartiles**						
Significant fibrosis	1.5232 (1.5224–1.5240)	< 0.001	1.5187(1.5179–1.5196)	< 0.001	1.3878 (1.3870–1.3886)	< 0.001
Advanced fibrosis	1.8500(1.8488–1.8511	< 0.001	1.7830 (1.7818–1.7841)	< 0.001	1.6910(1.6898–1.6921)	< 0.001
Cirrhosis	1.7358(1.7348–1.7369)	< 0.001	1.7594(1.7583–1.7605)	< 0.001	1.6567(1.6555–1.6578)	< 0.001
**ALS by clinical cut points**						
Significant fibrosis	1.6676(1.6666–1.6685)	< 0.001	1.6279(1.6269–1.6289)	< 0.001	1.4750(1.4740–1.4759)	< 0.001
Advanced fibrosis	1.9244(1.9230–1.9257)	< 0.001	1.8259(1.8246–1.8272)	< 0.001	1.6959(1.6946–1.6972)	< 0.001
Cirrhosis	1.7457(1.7446–1.7469)	< 0.001	1.7120(1.7109–1.7132)	< 0.001	1.5754(1.5742–1.5765)	< 0.001

### Subgroup analyses

**Fig. 2:**
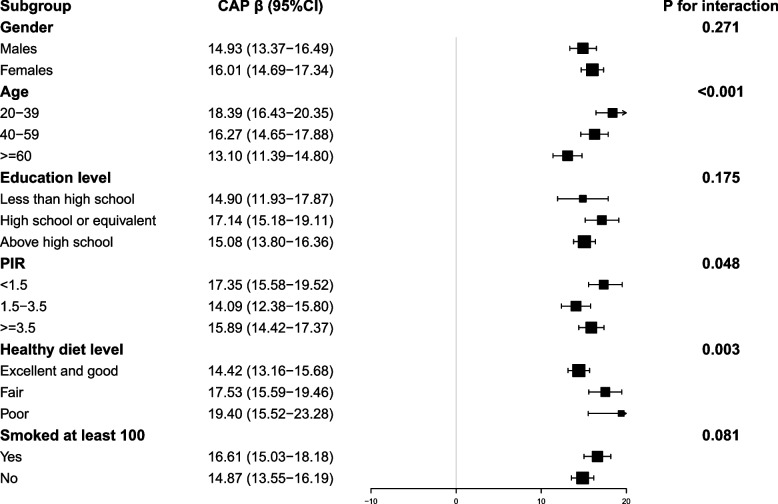
Subgroup Analysis for the Association between ALS and CAP

**Fig. 3:**
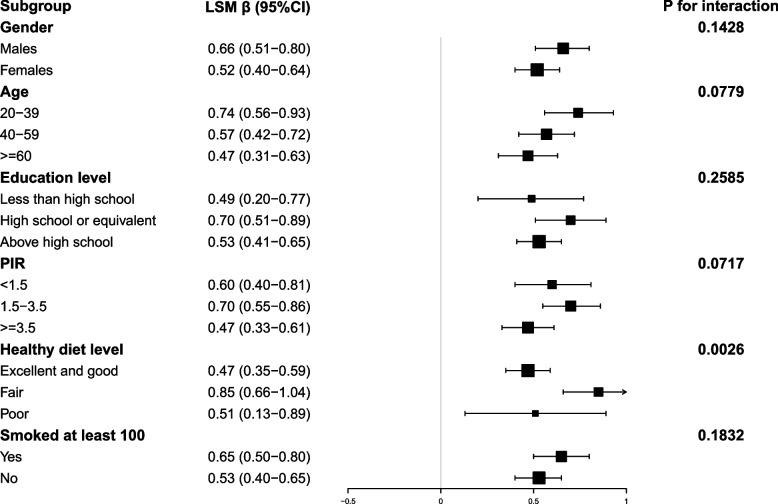
Subgroup Analysis for the Association between ALS and LSM

Figures 2 and 3 present subgroup analyses aimed at further evaluating the robustness of the correlation between ALS and CAP and LSM. The outcomes of our subgroup analysis demonstrate that this positive correlation remains consistent across diverse population settings (all *p* < 0.05). It was noted that in males, aged between 20–39, lower education levels, lower PIR values, poorer dietary habits, and higher smoking status, a significantly magnified correlation was evident between ALS and both CAP and LSM. Statistically, there was strong evidence of interaction effects between ALS and CAP across different age groups (*p* for interaction < 0.001), healthy diet levels (*p* for interaction = 0.003), and PIR (*p* for interaction = 0.048) categories. Additionally, a significant interaction effect was observed between ALS and LSM specifically within different healthy diet levels (*p* for interaction = 0.003). However, no significant interactions were observed within other predetermined subgroups (*p* for interaction > 0.05).

## Discussion

In this nationally representative study, ALS levels were positively correlated with the degree of hepatic steatosis and liver fibrosis, especially among young individuals aged 20–39, those with poorer dietary habits, and individuals with lower PIR values. Our study findings suggest that ALS levels serve as independent risk factors for monitoring the risk of hepatic steatosis and liver fibrosis.

To the best of our knowledge, this is the first study to assess the association between ALS and the degree of hepatic steatosis and liver fibrosis. Previous studies have demonstrated a significant association between stress and the incidence and mortality of liver disease [24, 25], particularly the role of the stress response in metabolic dysfunction-associated fatty liver disease [13]. A review by György described the utility of allostasis in non-alcoholic fatty liver disease from a molecular point of view, which was in line with our findings [12]. Yunzi Liu et al. constructed an experimental animal model of chronic stress and discovered that notwithstanding the chronicity of stress induces diminished dietary consumption and visceral adiposity, there is pronounced elevation in hepatic triglycerides and cholesterol levels, accompanied by augmented serum transaminase and inflammatory cytokine concentrations [26]. Czech et al. ascertained that mice subjected to a chronic stress model (congested communal living environment) displayed notable hepatic oxidative stress and inflammatory reactions in comparison to mice raised in a single housing environment when nourished with a normal diet [27]. Although heterogenous stress models were applied in these experimental inquiries, they collectively indicate a plausible correlation between the allostatic burden and the pathogenesis of hepatic steatosis and chronic inflammation.

Previous research has established a correlation between hepatic steatosis and fibrosis with subcomponents included in the ALS. Cardiovascular factors, such as dyslipidemia and hypertension, exhibit intricate interconnections with hepatic steatosis and fibrosis [28–30]. Metabolic factors like diabetes and high body mass index are causally linked to the development of hepatic steatosis [31, 32]. Lastly, systemic biomarkers of inflammation, including C-reactive protein levels, positively correlate with the severity of hepatic steatosis and fibrosis [31].

In our study, we did not explore potential associations between individual factors and hepatic steatosis or fibrosis in the adjusted analysis. Instead, chronic diseases typically extend their influence beyond singular biological functions or organs, encompassing the development of co-morbidities that impact overall outcomes. These comorbidities may arise from shared disease processes or diverse etiologies [12]. The concept of allostatic load offers a conceptual framework to comprehend the cumulative physiologic alterations inherent in chronic illnesses, demonstrating greater utility in stress evaluation and the appraisal of associated biological burden when compared to isolated biomarkers [11, 33]. Heightened allostatic load correlates with the advanced stages of ailments [11, 34]. As allostatic load gradually accumulates, it exacerbates deviation from the initial homeostatic set point, thus culminating in late-stage disease where physiological functions progressively deteriorate and become irreparable. Elevated allostatic load poses constraints not only on the likelihood of spontaneous remission but also on the efficacy of medical interventions. Consequently, applying the models of allostasis and allostatic load in the degree of liver steatosis and liver fibrosis may foster an enriched comprehension of disease progression, as well as facilitate the formulation of strategies encompassing prediction, prevention, and management.

Currently, the existing body of research on the association between allostatic load and human fatty liver disease is relatively scant, particularly when considering disease subgroups and interactions. Our subgroup analysis reveals that allostatic load impacts the severity of hepatic steatosis and fibrosis more pronouncedly in younger individuals with lower socio-economic status and subpar dietary health. This group may lack the requisite resources to cope with environmental stresses, which can result in the livers of such individuals working harder to maintain health and repair damage. This can lead to hepatic steatosis and excessive fibrosis, which in turn can exacerbate the severity of liver disease. Importantly, the SLD significantly correlates with the high prevalence of obesity and metabolic syndrome among young individuals, leading to a substantial escalation in disease occurrence over recent decades and substantially heightening the risk of liver cirrhosis and the necessity for liver transplantation [35]. Consequently, recognizing the allostatic load experienced by adolescents and young adults is of utmost importance. A systematic review by Jenny Guidi et al., encompassing 267 original investigations, suggested that factors such as low socioeconomic status, residing in impoverished communities, and indulging in unhealthy lifestyle habits (e.g., sedentariness, poor dietary patterns, and disturbed sleep) contributed to an augmented allostatic load [10]. Moreover, individual psychological well-being and coping mechanisms may moderate the association between social demographic influences and allostatic load. Nevertheless, the interpretation of these findings must be approached with caution due to the restricted nature of the sample size in this study and the potential influence of confounding factors. Consequently, further meticulously devised prospective studies are essential to gain a deeper understanding of this subject matter.

The exact mechanisms underlying the positive correlation between allostatic load and liver steatosis and fibrosis remain unclear. From an adaptive perspective, during sustained calorie surplus, excess fat is increasingly stored in non-adipose organs such as pancreatic cells, muscles, and the liver to accommodate the increased fat storage demands. Higher adaptation load can alter dietary habits, increase cravings for high-calorie foods, and lead to further fat accumulation to meet the body's increased demand for glucose levels [36]. Additionally, when the sympathetic nervous system (SNS) and hypothalamic–pituitary–adrenal (HPA) axis are chronically activated, a large number of catecholamines are released, which can promote lipolysis and lead to fat accumulation in the liver [37]. Furthermore, emotional stressors under high adaptation load can activate the hypothalamus to secrete various neurohormones, promoting the synthesis and secretion of glucocorticoids (GCs). Excessive levels of GCs have been shown to cause abnormal fat breakdown, resulting in the production of large amounts of free fatty acids, elevated blood glucose levels, and insulin resistance (IR) [14]. The continued presence of IR can disrupt hepatic lipid and glucose metabolism as well as systemic low-grade inflammation (manifested by increased levels of tumor necrosis factor-alpha (TNF-α), interleukin (IL)-1β, IL-6, and CRP) [38]. Moreover, stress and activation of the HPA axis can lead to gut dysbiosis, which is connected to liver steatosis, meaning the gut-liver axis [13]. Of considerable importance is the observation that elevated levels of oxidative stress in hepatic tissue stimulate additional adaptive responses that modulate the course of non-alcoholic fatty liver disease. Excessive lipid peroxidation can induce oxidative injury to genomic DNA, which is intricately linked to the pathogenesis of hepatocellular carcinoma. Lastly, it is imperative to underscore that allostatic load is intricately associated with an elevation of pro-inflammatory responses [39]. Potential mechanisms that contribute to this effect include dysbiosis and increased intestinal permeability in the gut-liver axis, as well as the intricate immunomodulatory effects of glucocorticoids and the development of resistance to corticosteroids [40]. However, this adaptive response is determined by various molecular pathways involved in regulating adipocyte differentiation, connective tissue growth, and angiogenesis, as well as their genetic driving factors.

Our study holds its strengths. Firstly, this study represents the first exploration of the association between ALS and the degree of liver steatosis and fibrosis in a large, representative sample of the US population. Secondly, the data employed in this study are derived from the NHANES, furnishing a sizable and representative sample with stringent quality control protocols and well-trained personnel meticulously collecting high-quality clinical variables, thus ensuring the reliability and validity of the findings. However, our study has some limitations. Firstly, due to its cross-sectional nature, this analysis lacks the ability to establish temporal order and ascertain causality. Secondly, the degree of hepatic steatosis and fibrosis in this study was assessed using transient elastography, while multiple studies have demonstrated its high diagnostic accuracy [41–43], liver tissue biopsy remains the gold standard. Thirdly, the calculation of allostatic load scores exhibits noteworthy variability, and despite our utilization of multiple approaches, the exclusion of certain biomarkers involved in the stress response may have potentially influenced the findings of the study. Future investigations should explore alternative algorithms for calculating ALS. However, by simultaneously incorporating clinical criteria and biological markers in evaluating ALS, our study will help elucidate the relationship between clinical and biological parameters and better identify states of excessive allostatic load than using either criterion alone. Fourth, the potential for bias inherent in the study design must be acknowledged. Given the limitations of the dataset, there is a possibility of selection bias and generalisability limitations in the results. Some of the covariates, such as dietary health level and physical activity data, were self-reported by participants through recall, which may have introduced a degree of memory bias. Lastly, despite rigorous adjustment for several pertinent confounding factors, the potential residual influence of other confounders cannot be entirely ruled out, necessitating cautious interpretation of the study findings. For instance, the long-term use of certain medications, such as antibiotics and non-steroidal anti-inflammatory drugs, is linked to an increased risk of hepatic steatosis and hepatic fibrosis. Conversely, the long-term use of glucose-lowering drugs is associated with a decreased risk of these conditions. Certain comorbidities, such as cardiovascular or other chronic diseases, and genetic factors can bias the results.

## Conclusion

The present study suggests that ALS levels are associated with the extent of liver steatosis and fibrosis, particularly within specific individual behavioral contexts. To validate our findings, larger-scale prospective investigations are warranted.

## Data Availability

The datasets presented in this study can be found in online repositories. The names of the repository/repositories and accession number(s) can be found at: https://wwwn.cdc.gov/nchs/nhanes.

## Abbreviations

ALP: Alkaline phosphatase
ALT: Alanine transaminase
ALS: Allostatic load score
AST: Aspartate transaminase
BMI: Body mass index
CRP: C-reactive protein
CAP: Controlled attenuation parameter
GGT: Gammaglutamyl transferase
HDL: High-density lipoprotein
HbA1c: Glycated hemoglobin
VCTE: Vibration-controlled transient elastography
LSM: Liver stiffness measurement
NHANES: National health and nutrition examination survey
PIR: Poverty income ratio
SLD: Steatotic liver disease
Tc: Total serum cholesterol
